# A Multicomponent Intervention to Train and Support Family Medicine Providers to Promote Pre-exposure Prophylaxis (PrEP) for Adolescent Girls and Young Women in the Deep South: Protocol for the PrEP-Pro Study

**DOI:** 10.2196/44908

**Published:** 2023-03-21

**Authors:** Oluwaseyi O Isehunwa, Samantha V Hill, Alex Tobias Menninger, Brook Hubner, Douglas Krakower, Dustin M Long, Madeline C Pratt, Meredith E Clement, Nicholas Van Wagoner, Robin Gaines Lanzi, Tina Simpson, Latesha Elopre, Lynn T Matthews

**Affiliations:** 1 Division of Infectious Diseases Department of Medicine University of Alabama at Birmingham Birmingham, AL United States; 2 Department of Pediatrics School of Medicine University of Alabama at Birmingham Birmingham, AL United States; 3 School of Public Health University of North Carolina at Chapel Hill Chapel Hill, NC United States; 4 Department of Medical Education University of Alabama at Birmingham Birmingham, AL United States; 5 Division of Infectious Diseases Department of Medicine Beth Israel Deaconess Medical Center Boston, MA United States; 6 Department of Biostatistics School of Public Health University of Alabama at Birmingham Birmingham, AL United States; 7 Section of Infectious Diseases Department of Medicine Louisiana State University Health Sciences Center New Orleans, LA United States; 8 Department of Health Behavior School of Public Health University of Alabama at Birmingham Birmingham, AL United States; 9 Tulane University School of Medicine New Orleans, LA United States

**Keywords:** pre-exposure prophylaxis (PrEP), training, PrEP Pro intervention, family medicine trainees, adolescent girls and young women (AGYW), family medicine, teens, young women, prevention, HIV, community, sexual health, reproductive health

## Abstract

**Background:**

Pre-exposure prophylaxis (PrEP) is a highly effective biomedical prevention intervention and a major strategy for reducing the HIV burden in the United States. However, PrEP provision and uptake remain lower than estimated needs, and in ways that may exacerbate HIV disparities among Black adolescent girls and young women in the southern United States. Data suggest that gaps in provider knowledge of HIV epidemiology and PrEP and skills assessing sexual health practices are important barriers to provision and uptake, with limited evidence-based interventions to address these gaps.

**Objective:**

This paper describes the “PrEP-Pro” intervention, a multicomponent intervention to train and support family medicine (FM) trainees to promote PrEP for adolescent girls and young women in Alabama.

**Methods:**

The PrEP-Pro intervention comprises 3 main components guided by the Capability-Opportunity-Motivation-Behavior (COM-B) model for behavioral change and the Consolidated Framework for Implementation Research (CFIR): (1) provider HIV epidemiology and PrEP education, (2) sexual history taking, and (3) PrEP Champions. In phase 1, we will work with community advisory boards (providers and clients) and then conduct focus groups with FM trainees to adapt content to train FM residents on HIV epidemiology and PrEP and develop implementation strategies, including provider-facing tools and client-facing educational materials. In phase 2, we will pretest and then pilot-test the initially adapted PrEP-Pro intervention with FM trainees. FM trainees will complete baseline, 3-, and 6-month questionnaires post PrEP-Pro intervention. We will also conduct in-depth interviews (IDIs) with FM pilot participants, adolescent girls and young women who accessed care after the PrEP-Pro pilot, and key stakeholders. The primary outcomes are PrEP-Pro acceptability and feasibility, which would be assessed using validated instruments at months 3 (among pretest participants) and 6 (among pilot participants). Secondary outcomes will also be assessed, including PrEP knowledge, sexual history-taking attitudes and practices, PrEP prescriptions among adolescent girls and young women encounters, and sexually transmitted infections (STIs) and HIV testing among adolescent girls and young women encounters in 6 months.

**Results:**

Study results will be disseminated to practices, state health officials, and other key stakeholders to solicit feedback on implementation opportunities and challenges to inform a hybrid effectiveness implementation trial. Our results will also be presented at local and national conferences and submitted to peer-reviewed journals.

**Conclusions:**

As PrEP grows, there is a pressing need to train FM providers and develop appropriate, contextually relevant tools to support PrEP implementation. The PrEP-Pro intervention is a multicomponent intervention to train FM residents across Alabama on sexual history-taking, PrEP provision for adolescent girls and young women, and supporting practice-based PrEP Champions. The PrEP-Pro intervention is anticipated to increase PrEP prescriptions for adolescent girls and young women and expand comprehensive sexual and reproductive health care for adolescent girls and young women in rural and urban Alabama.

**International Registered Report Identifier (IRRID):**

PRR1-10.2196/44908

## Introduction

In the United States, Black adolescent girls and young women are disproportionately affected by HIV compared to their White and Hispanic counterparts [1]. In 2019, Black adolescent girls and young women accounted for 54% of the approximately 36,801 new HIV diagnoses among women, despite representing just 13% of the female population [2]. Further estimates suggest that Black adolescent girls and young women are at disproportionately higher risk of HIV in the south than in other regions, where they account for 67% of new HIV diagnoses among women [3,4], with a growing number among those living in rural areas [4-6].

Pre-exposure prophylaxis (PrEP) is a highly effective biomedical prevention intervention and is an important strategy for ending the HIV epidemic in the United States. [7-9]. In 2012, the US Food and Drug Administration approved the first PrEP agent, a fixed oral combination of tenofovir disoproxil fumarate/emtricitabine for use in adults [10,11], and later broadened the approval to include adolescents in 2018 [12]. Most recently, in 2021, the Food and Drug Administration approved a long-acting injectable formulation, cabotegravir, as PrEP for adults and adolescents with vaginal or anal exposure to HIV [13].

Despite advances in biomedical PrEP therapeutics to reduce HIV incidence and increase patient autonomy, PrEP uptake remains low compared to estimated needs and in ways that may exacerbate disparities in HIV incidence [14,15]. In a cross-sectional study of 429 adolescents (aged 15-21 years) accessing preventive care in an urban adolescent primary care center in Alabama, almost half were PrEP-eligible, yet none were prescribed PrEP [16]. National data also suggest low PrEP prescription among adolescents and young women, as well as PrEP inequities by race and geographical location [14,17]. In 2019, only approximately 10% of women aged 16 years and older with indications for PrEP received a PrEP prescription [17]. Additionally, PrEP prescriptions among those with indications for PrEP were lowest among adolescents and young adults aged 16-24 years (16%) compared to adults aged 35-44 years (28%), and among Black individuals (8%) compared to White individuals (63%) [17]. Sullivan and colleagues [14] in a recent study using commercial pharmacy data to count PrEP users from 2012-2021 found the lowest PrEP use in the South compared to other US regions, and the lowest PrEP access in relation to the needs among the Black compared to the White individuals.

Multilevel barriers to PrEP provision and uptake include client-level barriers (such as low PrEP awareness, mistrust of the health care system, and PrEP-associated stigma) [18-21], health care system–level barriers (such as knowledge gaps and comfort among providers, unconscious bias, and racism) [22-24], and structural-level barriers (such as costs, reimbursement, and lack of health insurance) [24-26]. More specifically, in the South, PrEP provision and uptake have been limited by geographical factors—living in rural areas with limited access to PrEP providers [7], provider and health care system factors, including PrEP knowledge [27], provider sexual assessment skills [27], perceived provider racism [28], PrEP stigma [28,29], and health care capacity, among many other factors [7,30,31]. Thus, training providers in taking a sexual history and PrEP clinical care may be a successful strategy to increase PrEP use among Black adolescent girls and young women in the South [31-34].

Among health care providers (HCPs), family medicine (FM) practitioners have tremendous reach in rural communities and provide crucial preventive care for underserved populations [35]. They also provide transitional care as children advance into late adolescence, which often coincides with the onset of sexual activity [36-38]. In predominantly rural states like Alabama with high HIV incidence in rural communities, FM practitioners are a critical workforce to engage in HIV prevention methods like PrEP. Building the HIV workforce capacity is also responsive to the Ending the HIV Epidemic (EHE) national goals to improve our epidemic and decrease current inequities [39].

This paper outlines a protocol to develop and pilot a multicomponent intervention to train and support FM trainees to promote PrEP for adolescent girls and young women in Alabama. Our primary aims are to (1) adapt content to train FM trainees on HIV epidemiology and PrEP; (2) take a gender-neutral sexual history, and support “PrEP Champions,” (ie, FM trainees committed to promoting PrEP within the clinic); (3) pretest and pilot test the initially adapted intervention; and (4) conduct a formative evaluation to explore determinants of implementation. This proposed intervention provides an opportunity to expand comprehensive sexual and reproductive health care for adolescent girls and young women and train providers in HIV prevention.

## Methods

### Study Design and Setting

We are conducting an implementation science mixed methods study to develop and pilot test a multicomponent intervention—PrEP-Pro—for FM trainees in urban and rural Alabama to promote PrEP education, counseling, and prescription to Black adolescent girls and young women in Alabama. We chose to work with FM practitioners who provide comprehensive health care services across the lifespan, including adolescent girls and young women, and dominate the rural workforce [35]. In addition, we opted to work with trainees as provider behavior change may be more effective when integrated into the formative stages of training [40,41]. The FM program partners include trainees and providers from 4 programs across the state of Alabama, including programs based in rural and urban areas.

**Figure 1:**
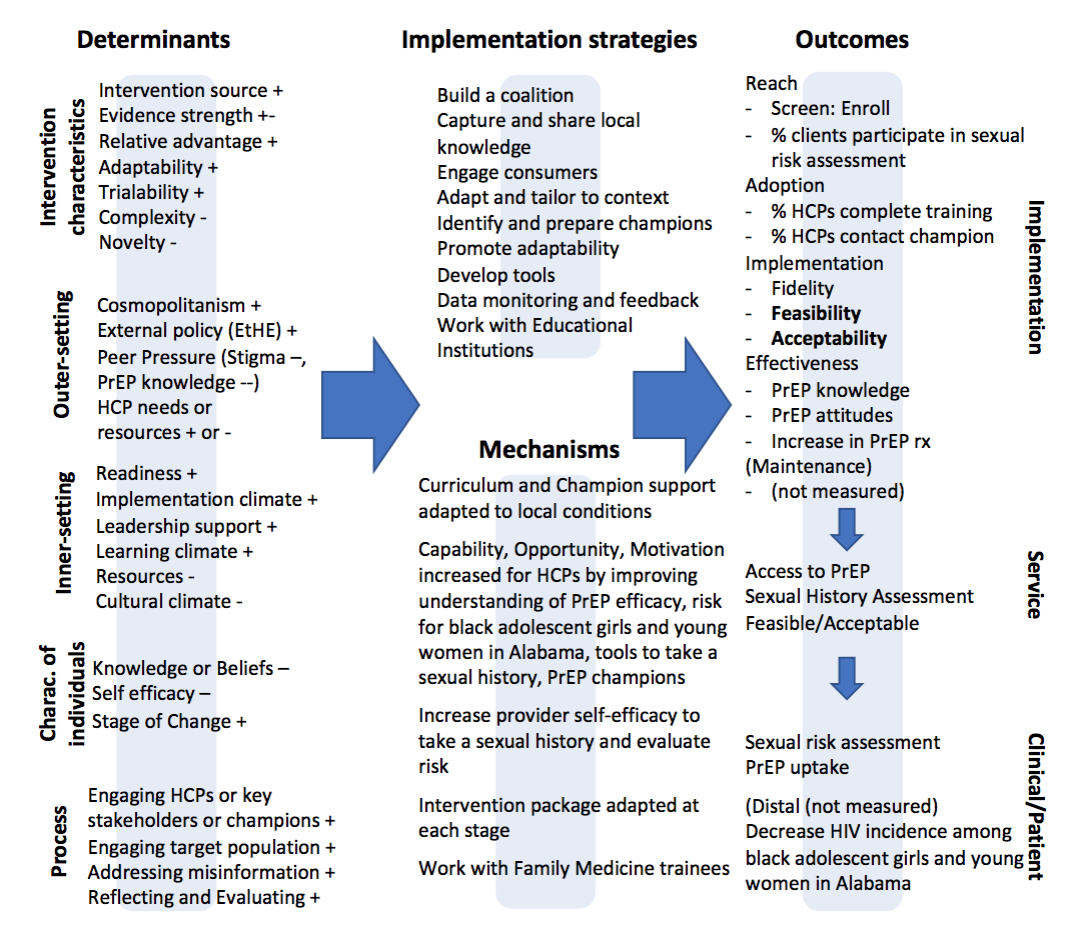
Implementation Research Logic Model (PrEP-Pro). The symbols “+" and “-" serve to indicate the “valence” of the determinants, or whether we anticipate the determinant will be a facilitator (+), a stronger facilitator (++), a barrier (-), a stronger barrier (—), or have a mixed impact *(+/-) on the implementation of the intervention. HCP: health care provider; PrEP: pre-exposure prophylaxis.

The PrEP-Pro intervention is theoretically informed by the Capability-Opportunity-Motivation-Behavior (COM-B) model for behavioral change [42] and the Consolidated Framework for Implementation Research (CFIR) [43]. The COM-B model for behavioral change proposes 3 components to any behavior [42]. Capability (C) refers to how capable providers feel about performing a behavior. For example, FM residents must be able to efficiently take a sexual history from adolescents to assess risk. Opportunity (O) refers to the social and physical opportunity to engage in the behavior. FM residents need support (eg, PrEP Champions) to navigate health insurance, privacy issues, and community stigma to offer PrEP. Motivation (M) refers to the desire to carry out the behavior in the face of other competing interests and may be addressed by helping FM residents understand the effectiveness of PrEP or by engaging PrEP Champions to motivate FM residents to prescribe PrEP. Our PrEP-Pro intervention addresses each component of the COM-B model to facilitate the increase of PrEP prescriptions to Black adolescent girls and young women. We also apply CFIR to consider multiple determinants required to increase PrEP prescriptions to Black adolescent girls and young women. CFIR is an implementation science determinant framework widely used to guide the systematic assessment of multilevel contextual factors that affect implementation and includes 5 domains, 48 constructs, and 19 subconstructs [43,44]. The 5 domains are (1) intervention characteristics or innovation (ie, the product or the “thing” being implemented), (2) inner setting (ie, setting where the innovation is being carried out), (3) outer setting (ie, setting where the inner setting exists), (4) individuals (ie, roles and responsibilities of individuals), and (5) implementation process (ie, various activities carried out to implement the innovation) [44]. For example, the individual-level determinants at the provider level that may influence PrEP prescription include knowledge and beliefs around PrEP and conducting a sexual history or risk assessment, and self-efficacy as identified in our studies and also supported by prior literature [21,22,24,31,45]. The inner setting determinants include readiness for change, ease of access to knowledge and information about sexual history taking and PrEP, and leadership engagement within FM training programs to innovate and provide research opportunities for residents. The outer setting determinants include state-level EHE impetus and PrEP products in the pipeline. Therefore, guided by CFIR, we will draft and further explore and understand more granularly the implementation determinants of PrEP-Pro intervention to inform a future hybrid effectiveness/implementation trial (Figure 1).

### PrEP-Pro Multicomponent Intervention

The PrEP-Pro intervention consists of 3 main components. Tailored training in HIV epidemiology and PrEP basics, sexual history taking, and support practice–based PrEP Champions will be provided. We will first adapt and develop the PrEP-Pro intervention and then conduct a pilot test. To guide the systematic development of our intervention, implementation, and evaluation, we will use intervention mapping (IM) techniques [46,47].

### Phase 1: Development of the Multicomponent Intervention

#### Overview

**Figure 2:**
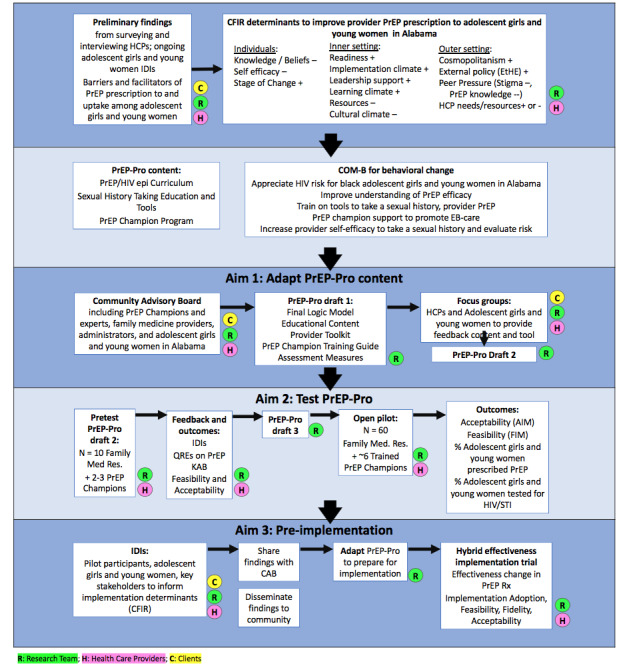
PrEP-Pro study flow and aims. AIM: Acceptability of Intervention Measure; CAB: community advisory board; CFIR: Consolidated Framework for Implementation Research; FIM: Feasibility of Intervention Measure; HCP: health care provider; IDI: in-depth interview; PrEP: pre-exposure prophylaxis; PrEP KAB: pre-exposure prophylaxis knowledge, attitudes, and behavior; QRE: questionnaire; STI: sexually transmitted infection.

In phase 1, we will develop a multicomponent intervention, PrEP-Pro intervention (Figure 2). This includes the adaptation of content to train on HIV epidemiology and PrEP and the development of implementation strategies including provider-facing tools and client-facing educational materials. We plan to make the content developed compatible with the Project Extension for Community Healthcare Outcomes to optimize dissemination opportunities [48]. Project Extension for Community Healthcare Outcomes is a disease management approach using case-based web-based learning to increase knowledge among providers and standardize best practices for the screening, care, and treatment of a disease [49,50].

#### Anticipated Adaptations

We plan to make adaptations or develop new content in the following areas: (1) epidemiology of HIV risk for Black adolescent girls and young women in Alabama; (2) PrEP indications for adolescent girls and young women; (3) PrEP knowledge, attitudes, and behavior (KAB) tool; (4) sexual history taking with focus on adolescent girls and young women needs and nuances in how to ask questions to this age group; (5) sexual history screening tool for adolescent girls and young women including strategies to optimize sexual behavior screening for adolescent girls and young women; and (6) PrEP champion toolkit.

#### Adaptation Process

##### Review of Existing Literature

To adapt content, the research team reviewed existing literature and web-based databases to identify relevant and more recent content on sexual history taking [51-54], HIV epidemiology [55-57], PrEP curriculum [58-61], local resources and tools for PrEP Champions, as well as relevant instruments to assess PrEP knowledge, attitudes, and beliefs [62,63]. The lead authors of publications were contacted through email to request additional materials discussed in their publications which are not accessible digitally.

##### Review With the Scientific Advisory Board (SAB)

The research team worked with a broader scientific investigative team inclusive of expertise in adolescent health, medical education, FM training, and HIV prevention or PrEP care to solicit input and review materials and data gathered from the review of existing literature.

##### Community Engagement With the Community Advisory Board (CAB)

Drawing on the principles of cocreation [64,65], we developed 2 CABs, 1 group (N=7) comprising FM representatives from rural and urban settings, including FM trainees, administrators, and advocates, and a second group (N=4) comprising Black adolescent girls and young women. The CABs were convened separately due to the different roles, expertise, and comfort level in sharing. CAB members were identified and recruited based on recommendations from our scientific team and our collaborating FM programs. CAB members meet remotely for 1 hour every month to review and reach a consensus on the content of the curriculum and tools that address adolescent tenets, tools to measure PrEP knowledge, as well as provide input on optimal strategies to train FM residents and promote or offer PrEP. This process is ongoing, and details on the iterations made to the content of the curriculum and tools will be published in a subsequent paper. In addition to working with the CAB to adapt content, we also plan to work with the CAB throughout the study to help with the interpretation of results and dissemination of study findings.

##### Focus Group Discussions (FGDs) With FM Providers (Trainees)

Two FGDs comprising FM trainees will be conducted (December 2022-January 2023) to seek feedback on the draft PrEP-Pro content and a newly developed PrEP KAB tool. Provider participant eligibility criteria include (1) being a licensed HCP (MD, DO) trainee, (2) providing medical care to adolescent girls and young women in Alabama, (3) working in a clinic setting where at least 20% of the population is Black, and (4) being able or willing to provide informed consent. Participants will complete a brief questionnaire outlining sociodemographic and clinic practice characteristics. During the FGD, the facilitator will present the initial adapted PrEP-Pro intervention (including the initial draft of the PrEP-Pro content, badges, flow charts, and tools) iteratively to the 2 groups of 4-6 FM trainees. FGDs will be conducted virtually or in an enclosed private location and will last for approximately 60 to 90 minutes. Trained interviewers will facilitate all FGDs, including probing, asking open-ended questions, ensuring nonjudgmentalism, and using grounded theory. FGDs will be digitally recorded and transcribed. All FGD participants will be reimbursed US $50 for their time.

### Phase 2: Testing the Initially Adapted PrEP-Pro Intervention

#### Overview

Using peer champions to improve provider knowledge and quality of care is an evidence-based implementation strategy [66]. Studies describe the presence of change champions within clinical practices as essential facilitators of quality improvement [67], and such champions can play an essential role in communication, marketing, education, skill-building, and assessment [68]. Provider champions are agents of change [69], empowering their peer providers within their practice to engage in innovations, practice new skills, and implement new care strategies with more ease and confidence.

The primary aim of phase 2 is to pretest and pilot-test the initially adapted PrEP-Pro intervention with FM trainees in Alabama (Figure 2). We will pretest the intervention by training 2 FM trainees as PrEP champions with the initially drafted curriculum, who will then deliver the draft curriculum and ongoing support to 8-12 FM trainees (from at least 1 urban and 1 rural setting). In addition to receiving additional training and support, PrEP champions will have access to PrEP consultants and experts.

The pretest phase will allow us to optimize the PrEP-Pro intervention (refine the number and length of sessions, educational material content, and other details that will be key to maximizing acceptability, feasibility, and impact). Thereafter, an open pilot trial of the PrEP-Pro intervention will be conducted by training an additional 6 FM trainees to serve as PrEP Champions and enrolling 60 unique FM trainees from rural and urban settings into the study.

Training PrEP Champions can thus support entire practices to sustain the provision of evidence-based care, navigate medical assistance programs and insurance coverage and troubleshoot new challenges [70].

#### PrEP-Pro Champion Training Workshop

PrEP Champions will complete an estimated 4 hours of training over 3 sessions using the PrEP Champion training tools. PrEP Champions will receive more comprehensive detailed training on HIV epidemiology and how to remain updated with the latest data, how to use the adapted sexual history-taking tool, PrEP efficacy, and indications. They will also be supported by an established PrEP Champion on our team to train on and role play, prescribing requirements, nuances to prescribing and fielding questions from adolescents, PrEP adherence support, medical assistance program navigation, and effective curriculum delivery to peers. An education expert member of the scientific team will coach PrEP Champions in effective curriculum delivery. PrEP Champions will also be trained to troubleshoot challenges that may arise and will be encouraged to set up a “community of practice” within their group for check-ins regarding challenges and solutions (eg, through a monthly call or WhatsApp or GroupMe chat). PrEP Champions will also log their time and provider contacts through a web-based app (eg, toggl.com, myhours.com, or text the project coordinator) over 3 months (pretest) and 6 months (pilot). To optimize PrEP-Pro intervention fidelity, PrEP Champions will complete standardized training and use structured protocols. Early training sessions will be monitored. Subsequently, a subset of sessions (at least one additional session per champion) will be audio-recorded and reviewed by the investigators, and a checklist will be developed for a protocol adherence. This will allow for real-time feedback and minimize drift in the approach [71]. After the initial training, PrEP Champions will complete an evaluation to assess HIV epidemiology, PrEP knowledge, and the acceptability and feasibility of training curricula.

#### Provider PrEP Education

Other FM trainees will receive 2 hours of case-based real-time sessions with a videoconference hosted by a local PrEP Champion including a 1-hour session on PrEP prescription and a 1-hour session on sexual history taking.

#### Participants and Eligibility Criteria

Participants will be identified and purposefully selected through FM partner program directors and faculty. The project coordinator will work with site leadership to generate a list and contact details for all practicing FM trainees at the study sites. A random-order list of eligible FM trainees stratified by gender, race, training site, year of training, and age quartiles will be generated. FM trainees will be approached by the project coordinator (via email, phone, or in person) and invited to participate in the pilot program. Reasons for declining will be recorded. Eligibility for PrEP Champions, pretest, and pilot participants will include (1) a licensed HCP (MD, DO) trainee, (2) providing medical care to adolescent girls and young women in Alabama, (3) working in a clinic where at least 20% of the population is Black, and (4) being able or willing to provide informed consent. The aim is to ensure that trainees feel free from coercion by including at least 1 trainee in our CAB. All language within the recruitment materials and consent forms will be explicit that participation in this study is not mandatory nor impacts trainee clinical obligations, clinical evaluations, or standing within the residency program. Participants will be reimbursed for their time.

#### Primary Outcomes

The primary outcomes to be assessed per our original protocol are PrEP-Pro acceptability and feasibility (Table 1). However, as we engage our CAB and SAB continually and iteratively, these outcomes are subject to evolve based on feedback.

**Table 1:** Primary and secondary outcomes.

Outcomes and measures			Source of data
**Primary outcomes**			
	Acceptability of Intervention Measure (AIM): 4 items, 5-point Likert scale Intervention Appropriateness Measure (IAM): 4 items, 5-point Likert scale Evidence-Based Practice Attitude Scale (EPAS-15): 15 items, 5-point Likert scale Feasibility of Intervention Measure: 4 items, 5-point Likert scale Number screened, eligible, and enrolled Number or proportion who completes all planned training modules Number or proportion who contact the PrEP^a^ Champion at least once after training	Quantitative data (Survey/Process measures)	
	PrEP-Pro Feasibility PrEP-Pro Acceptability Willingness to prescribe PrEP	Qualitative data (IDIs^b^)	
**Secondary outcomes**			
	PrEP knowledge HIV epidemiology knowledge Sexual history taking beliefs and attitudes Willingness to prescribe PrEP	Quantitative data (Survey)	
	Sexual history taking practices HIV and STI^c^ tests or adolescent girls and young women encounters PrEP prescriptions or adolescent girls and young women encounters	Quantitative data (EMR^d^)	

^a^PrEP: pre-exposure prophylaxis.

^b^IDI: in-depth interview.

^c^STI: sexually transmitted infection.

^d^EMR: electronic medical record.

#### PrEP-Pro Acceptability

This will be assessed using three validated instruments: (1) Acceptability of Intervention Measure (AIM), a 4-item instrument with 5-point Likert scale response options [72], (2) Intervention Appropriateness Measure, a 4-item instrument with 5-point Likert scale response options [72], and (3) Evidence-Based Practice Attitude Scale, a 15-item instrument with 5-point Likert scale response options [73].

#### PrEP-Pro Feasibility

This will be assessed using the Feasibility of Intervention Measure, a 4-item instrument with 5-point Likert scale response options [72]. Additionally, to assess PrEP-Pro feasibility, the number of residents screened, eligible, and enrolled, the time to complete training, the number of participants who complete training, and the number of participants who contact PrEP champions at least once in the quarter after the training (as reported by each PrEP Champion via study logs) will also be described.

To assess these outcomes, FM trainees will complete questionnaires at 0, 3, and 6 months post PrEP-Pro training. Enrolled FM trainees (PrEP Champions, providers who pretest PrEP-Pro, and pilot participants) will complete a baseline questionnaire to capture sociodemographic data (age, race, gender, years of practice, and client volume), PrEP KAB practices, and experiences with PrEP provision. PrEP Champions and providers that pretest PrEP-Pro will complete a posttraining or intervention questionnaire within 3 months of training or 6 months of training for pilot participants. Questionnaires will be completed in REDCap by the participants via a secure link sent to their computer, phone, or other electronic devices.

#### Secondary Outcomes

Other outcomes will also be assessed including PrEP knowledge, sexual history taking attitudes and practices [74], PrEP prescriptions at adolescent girls and young women encounters, and sexually transmitted infection (STI) and HIV testing at adolescent girls and young women encounters (Table 1). We aim to explore how to optimize PrEP-Pro to maximize acceptability, feasibility, and impact including barriers and facilitators to PrEP-Pro initially adapted intervention.

To assess these secondary outcomes, data will be extracted from electronic medical records at baseline, 3 months, and 6 months after enrollment to provide data on the number of PrEP prescriptions to adolescent girls and young women, HIV testing, and STI testing among adolescent girls and young women in the 6 months before or after PrEP-Pro training. Additionally, IDIs will be conducted with PrEP Champions and pretest participants within 3 months of scheduled training completion. Interview guides will be developed using our conceptual frameworks, a recent review of literature, and input from study team members and the CAB and will focus on challenges, successes, barriers, and facilitators to PrEP prescription to adolescent girls and young women and feasibility, acceptability, anticipated impacts of the draft PrEP-Pro intervention. To plan for the implementation of PrEP-Pro as an intervention strategy to promote PrEP prescription, IDIs will be conducted with 25 pilot participants, 25 adolescent girls and young women who accessed care after the PrEP-Pro pilot, and ~12 other key stakeholders (eg, administrators and support staff) to explore anticipated determinants of implementation based on CFIR [43,75]. PrEP Champions will inform process-level domains, PrEP-Pro pilot providers and adolescent girls and young women will inform individual-level domains, and key stakeholders will inform outer-setting domains. PrEP-Pro will be updated based on any interval innovations in PrEP implementation science and present the findings to the CAB and consultants to seek feedback on implementation determinants. Interviews will be conducted remotely or in an enclosed private location and will last for approximately 45 to 60 minutes. All interviews will be digitally recorded and transcribed.

#### Sample Size Justification

The plan to pretest PrEP-Pro with 12 providers is consistent with recommendations for pretesting from IM literature [76] and our pretesting sample in prior intervention development. For this pilot study, the importance of sample size lies in establishing acceptability and feasibility and the precision of estimated changes in PrEP prescriptions. There will be a recruitment of 60 FM trainees throughout Alabama. With our sample, we can estimate this measure with a 95% margin of error of 0.26 SD, where SD is the observed standard deviation of the Feasibility of Intervention Measure or AIM score. For example, if we observe an SD of 5, the 95% CI would be ±1.3. Similarly, the margin of error for the change in average PrEP prescriptions 6 months before to 6 months after the intervention is ±0.26 SD.

### Ethical Considerations

Ethical approval for phase 1 of the study to assemble a CAB and conduct FGDs was approved by the Institutional Review Board (IRB) at the University of Alabama at Birmingham, Alabama (IRB-300008567). Written informed consent will be obtained from all FGD participants prior to their participation in the study. Both FGD participants and CAB members are compensated US $50 for their time (eg, an hour-long CAB meeting or an hour-long FGD). A separate ethics approval will be obtained from the institutional review board prior to commencing phase 2 of the study. All data will be kept confidential and stored in password-protected software with encryption. Only deidentified data will be reported.

### Data Analysis

#### FGD Analysis

FGDs will be audio recorded, transcribed, and initially analyzed using a structured template and memo-based approach with subsequent transcription and coding [77]. This template approach will consist of team members reviewing the recordings of the FGD, making notes of important themes and responses, and then summarizing the interview content. Using this method, the research team will be able to quickly discuss and apply key data points to the implementation strategy design, and later, a more in-depth analysis of the transcribed discussions will be conducted using content analysis [78,79].

#### IDI Analysis

IDIs will be audio recorded, transcribed, and analyzed more in-depth using the content analysis approach [78,79].

Two investigators will independently review transcripts to generate an overarching thematic framework for data interpretation. Initial themes will be identified based on the initial readings of a subset of transcripts and guided by our theories or conceptual frameworks (COM-B and CFIR). Themes will be discussed among the scientific team, transcripts will be reread, and themes will be refined based on further review of the transcripts until saturation is achieved. Final themes including major themes and subthemes will be developed. A codebook will be developed with a detailed description of major themes and subthemes. The investigators will compare their thematic frameworks for consistency, and any discrepancies will be discussed until there is agreement on the thematic framework. Using multiple coders also enhances the validity of the analysis [80]. Kappa statistics will be computed to measure agreement between coders (interrater reliability test). Data will be reexamined, messages will be extracted and highlighted, and the ongoing discussion between coders will allow for further theorizing and interconnections between research questions, coding categories, and raw data (108). Emerging themes will be explored with the CAB and the SAB to enhance the validity of the findings. We will also compare data findings with results from our quantitative data and external review of the literature for consistency. NVivo (QSR International) software will be used to organize data and facilitate analyses.

### Statistical Analysis

Descriptive statistics will be calculated to characterize acceptability and feasibility measures collected 3 months (PrEP champions and pretest participants) and 6 months (pilot participants) after enrollment. All scores will be summarized using appropriate descriptive statistics. The number of participants screened, eligible, and enrolled; the proportion of participants who complete all planned training modules; and the proportion of participants who contact the PrEP Champion at least once in the quarter after training will be described. We will also describe the data and compare pre- and post-PrEP prescription or tests per adolescent girls and young women visit with nonparametric measures. Agreement between pre– and post–PrEP-Pro tests for categorical measures will be assessed using the McNemar test bivariate and using multiple logistic regression of post–PrEP-Pro outcomes adjusting for pre–PrEP-Pro measures and covariates. Covariates in adjusted models will include individual providers (such as age and race) and selected setting variables (such as client demographics, number of clients seen per day, and environment toward PrEP). Poisson regressions will determine associations between post–PrEP-Pro count measures (such as the number of PrEP prescriptions) and pre–PrEP-Pro counts, both unadjusted and covariate-adjusted. Similarly, for continuous outcomes (such as percent of adolescent girls and young women with PrEP prescription), Wilcoxon signed rank tests and linear models of post–PrEP-Pro measures would be used. We will examine missing data, but as our primary outcomes are feasibility or acceptability in which missing data (no response) is informative, no adjustments will be made.

## Results

Study results will be presented to the CAB members and disseminated to practices, state health officials, and other key stakeholders to solicit feedback on implementation opportunities and challenges to inform a hybrid effectiveness implementation trial. The results of this study will also be presented at local and national conference meetings and submitted to peer-reviewed journals for publication.

## Discussion

### Principal Findings

This protocol paper describes a research study to develop and pilot a theoretically driven multicomponent intervention, PrEP-Pro, to train and support FM trainees across Alabama on HIV epidemiology, sexual history taking, and PrEP provision for adolescent girls and young women. Although PrEP is a highly effective biomedical intervention that can reduce HIV incidence among adolescent girls and young women, uptake remains low among adolescent girls and young women populations due to several multilevel factors [8,14]. In Alabama, which has been identified in the EHE strategy for the United States as a geographic hot spot due to high rural incidence, almost half of new HIV infections occur in those aged 13-29 years [57]. As PrEP options expand, there is an urgent need for appropriate, contextually relevant training tools to support PrEP implementation. It is anticipated that the PrEP-Pro intervention will result in an increase in PrEP prescriptions for adolescent girls and young women and an expansion of comprehensive sexual and reproductive health care for adolescent girls and young women in rural and urban Alabama.

To our knowledge, this is the first provider intervention that seeks to promote PrEP prescription among adolescent girls and young women in the South. Working with FM trainees is an innovative approach to promoting PrEP provision among clinicians who work with adolescents in rural communities across the United States [35,81]. Adapting PrEP-Pro to trainees provides an opportunity to modify clinician behavior at an early stage of training and help seed the rural health workforce with effective HIV prevention advocates and providers. Combining education specialists in theory-based IM with community engagement allows us to adapt interventions with greater content retention and implementation.

Despite our proposed novel intervention, we acknowledge several limitations. Many PrEP uptake barriers in Alabama are structural, including structural racism, a lack of comprehensive education in school settings, costs to access repeated HIV or STI testing, transportation, and a lack of Medicaid expansion [7,25,26]. While our study aims to target provider-level barriers, delivering multilevel interventions to address client, provider, and structural-level barriers would be ideal. However, structural issues are addressed in other projects our group supports [82-84]. Another limitation is that our study focuses primarily on adolescent girls and young women, and our outcomes will assess PrEP prescriptions for adolescent girls and young women, such that our study findings may not be generalizable to all populations. However, we have developed our PrEP-Pro content and intervention so that FM trainees can support and prescribe PrEP to all populations. Additionally, our PrEP-Pro intervention provides content mainly on tenofovir disoproxil fumarate/emtricitabine PrEP agents, with some content on newer PrEP products (injectable PrEP). Further, there is the possibility of selection bias in those who choose to become PrEP Champions or engage in the pretest and pilot. Lastly, as this is a pilot study, there will be a lack of data to assess long-term changes in outcomes. Future prospective cohort studies with longer periods to assess implementation outcomes and effectiveness outcomes among adolescent girls and young women populations may be warranted.

### Conclusions

In conclusion, this study applies implementation and health services research science to adapt strategies to increase provider PrEP prescribing to adolescent girls and young women in the southern United States. As PrEP options expand, there is a pressing need to train FM practitioners and develop appropriate, contextually relevant training tools to support PrEP implementation. Study findings on the acceptability and feasibility of our PrEP-Pro intervention will help to inform a future hybrid effectiveness-implementation trial.

## Abbreviations

AIM: Acceptability of Intervention Measure
CAB: community advisory board
CFIR: Consolidated Framework for Implementation Research
COM-B: Capability-Opportunity-Motivation-Behavior
EHE: Ending the HIV Epidemic
FGD: focus group discussion
FM: family medicine
HCP: health care provider
IDI: in-depth interview
IM: intervention mapping
KAB: knowledge, attitudes, and behavior
PrEP: pre-exposure prophylaxis
SAB: scientific advisory board
STI: sexually transmitted infection

## Data Availability

Deidentified quantitative data sets generated during the study will be uploaded into a data-sharing site to be determined by the research team when the data are cleaned and analyzed. Due to the small sample size and difficulties in redacting qualitative data collected from a small group (FM providers from select programs in a small state), qualitative data with providers will not be shared.

## References

[1] HIV in the United States and dependent areas. Centre for Disease Control and Prevention.

[2] HIV and women: HIV diagnoses. Centre for Disease Control and Prevention.

[3] (2018). HIV surveillance report, 2017. Centre for Disease Control and Prevention.

[4] (2019). HIV in the Southern United States. Centre for Disease Control and Prevention.

[5] Piper K, Enah C, Daniel M (2014). Black southern rural adolescents' HIV stigma, denial, and misconceptions and implications for HIV prevention. J Psychosoc Nurs Ment Health Serv.

[6] Patel D, Taylor-Aidoo N, Marandet A, Heitgerd J, Maciak B (2019). Assessing differences in CDC-funded HIV testing by urbanicity, United States, 2016. J Community Health.

[7] Sullivan PS, Mena L, Elopre L, Siegler AJ (2019). Implementation strategies to increase PrEP uptake in the South. Curr HIV/AIDS Rep.

[8] Fonner VA, Dalglish SL, Kennedy CE, Baggaley R, O'Reilly KR, Koechlin FM, Rodolph M, Hodges-Mameletzis I, Grant RM (2016). Effectiveness and safety of oral HIV preexposure prophylaxis for all populations. AIDS.

[9] Riddell J, Amico KR, Mayer KH (2018). HIV preexposure prophylaxis: a review. JAMA.

[10] (2012). CDC statement on FDA approval of drug for HIV prevention. Centre for Disease Control and Prevention.

[11] Tanner MR, Miele P, Carter W, Valentine SS, Dunville R, Kapogiannis BG, Smith DK (2020). Preexposure prophylaxis for prevention of HIV acquisition among adolescents: clinical considerations, 2020. MMWR Recomm Rep.

[12] US Food and Drug Administration approves expanded indication for Truvada (emtricitabine and tenofovir disoproxil fumarate) for reducing the risk of acquiring HIV-1 in adolescents? First agent indicated for uninfected adolescents at risk of acquiring HIV. Gilead.

[13] (2021). FDA approves first injectable treatment for HIV pre-exposure prevention. US Food and Drug Administration.

[14] Sullivan P, Whitby S, Hipp P (2012). Trends in PrEP inequity by race and census region, United States, 2012-2021. https://www.natap.org/2022/IAC/IAC_26.htm.

[15] Siegler AJ, Bratcher A, Weiss KM, Mouhanna F, Ahlschlager L, Sullivan PS (2018). Location location location: an exploration of disparities in access to publicly listed pre-exposure prophylaxis clinics in the United States. Ann Epidemiol.

[16] Hill SV, Westfall AO, Coyne-Beasley T, Simpson T, Elopre L (2020). Identifying missed opportunities for human immunodeficiency virus pre-exposure prophylaxis during preventive care and reproductive visits in adolescents in the deep South. Sex Transm Dis.

[17] Monitoring selected national HIV prevention and care objectives by using HIV surveillance data United States and 6 dependent areas, 2020. Centre for Disease Control and Prevention.

[18] Calabrese SK, Dovidio JF, Tekeste M, Taggart T, Galvao RW, Safon CB, Willie TC, Caldwell A, Kaplan C, Kershaw TS (2018). HIV pre-exposure prophylaxis stigma as a multidimensional barrier to uptake among women who attend planned parenthood. J Acquir Immune Defic Syndr.

[19] Tekeste M, Hull S, Dovidio JF, Safon CB, Blackstock O, Taggart T, Kershaw TS, Kaplan C, Caldwell A, Lane SB, Calabrese SK (2019). Differences in medical mistrust between black and white women: implications for patient-provider communication about PrEP. AIDS Behav.

[20] Smith DK, Toledo L, Smith DJ, Adams MA, Rothenberg R (2012). Attitudes and program preferences of African-American urban young adults about pre-exposure prophylaxis (PrEP). AIDS Educ Prev.

[21] Pratt MC, Jeffcoat S, Hill SV, Gill E, Elopre L, Simpson T, Lanzi R, Matthews LT (2022). "We feel like everybody's going to judge us": Black adolescent girls' and young women's perspectives on barriers to and opportunities for improving sexual health care, including PrEP, in the Southern U.S. J Int Assoc Provid AIDS Care.

[22] Petroll AE, Walsh JL, Owczarzak JL, McAuliffe TL, Bogart LM, Kelly JA (2017). PrEP awareness, familiarity, comfort, and prescribing experience among US primary care providers and HIV specialists. AIDS Behav.

[23] Calabrese SK, Earnshaw VA, Underhill K, Hansen NB, Dovidio JF (2014). The impact of patient race on clinical decisions related to prescribing HIV pre-exposure prophylaxis (PrEP): assumptions about sexual risk compensation and implications for access. AIDS Behav.

[24] Hill SV, Pratt MC, Elopre L, Smith TV, Simpson T, Lanzi R, Matthews LT (2022). "Let's take that [stop sign] down." Provider perspectives on barriers to and opportunities for PrEP prescription to African American girls and young women in Alabama. AIDS Care.

[25] Mayer KH, Agwu A, Malebranche D (2020). Barriers to the wider use of pre-exposure prophylaxis in the United States: a narrative review. Adv Ther.

[26] Bonacci RA, Smith DK, Ojikutu BO (2021). Toward greater pre-exposure prophylaxis equity: increasing provision and uptake for Black and Hispanic/Latino individuals in the U.S. Am J Prev Med.

[27] Moore E, Kelly SG, Alexander L, Luther P, Cooper R, Rebeiro PF, Zuckerman AD, Hargreaves M, Bourgi K, Schlundt D, Bonnet K, Pettit AC (2020). Tennessee healthcare provider practices, attitudes, and knowledge around HIV pre-exposure prophylaxis. J Prim Care Community Health.

[28] Cahill S, Taylor SW, Elsesser SA, Mena L, Hickson D, Mayer KH (2017). Stigma, medical mistrust, and perceived racism may affect PrEP awareness and uptake in black compared to white gay and bisexual men in Jackson, Mississippi and Boston, Massachusetts. AIDS Care.

[29] Arnold T, Brinkley-Rubinstein L, Chan PA, Perez-Brumer A, Bologna ES, Beauchamps L, Johnson K, Mena L, Nunn A (2017). Social, structural, behavioral and clinical factors influencing retention in Pre-Exposure Prophylaxis (PrEP) care in Mississippi. PLoS One.

[30] Hing E, Hsiao CJ (2014). State variability in supply of office-based primary care providers: United States, 2012. NCHS Data Brief.

[31] Pratt MC, Hill SV, Elopre L, Simpson T, Lanzi R, Matthews LT (2022). PrEP prescription for black adolescent girls and young women in Alabama: findings from a survey of healthcare providers. J Int Assoc Provid AIDS Care.

[32] Blumenthal J, Jain S, Krakower D, Sun X, Young J, Mayer K, Haubrich R, CCTG 598 Team (2015). Knowledge is power! increased provider knowledge scores regarding pre-exposure prophylaxis (PrEP) are associated with higher rates of PrEP prescription and future intent to prescribe PrEP. AIDS Behav.

[33] Sales JM, Cwiak C, Haddad LB, Phillips A, Powell L, Tamler I, Sheth AN (2019). Brief report: impact of PrEP training for family planning providers on HIV prevention counseling and patient interest in PrEP in Atlanta, Georgia. J Acquir Immune Defic Syndr.

[34] Clement ME, Seidelman J, Wu J, Alexis K, McGee K, Okeke NL, Samsa G, McKellar M (2018). An educational initiative in response to identified PrEP prescribing needs among PCPs in the Southern U.S. AIDS Care.

[35] Grumbach K, Hart LG, Mertz E, Coffman J, Palazzo L (2003). Who is caring for the underserved? A comparison of primary care physicians and nonphysician clinicians in California and Washington. Ann Fam Med.

[36] Rand CM, Goldstein NPN (2018). Patterns of primary care physician visits for US adolescents in 2014: implications for vaccination. Acad Pediatr.

[37] King BM, Marino LE, Barry KR (2018). Does the Centers for Disease Control and Prevention's youth risk behavior survey underreport risky sexual behavior?. Sex Transm Dis.

[38] Kann L, McManus T, Harris WA, Shanklin SL, Flint KH, Hawkins J, Queen B, Lowry R, Olsen EO, Chyen D, Whittle L, Thornton J, Lim C, Yamakawa Y, Brener N, Zaza S (2016). Youth risk behavior surveillance - United States, 2015. MMWR Surveill Summ.

[39] Fauci AS, Redfield RR, Sigounas G, Weahkee MD, Giroir BP (2019). Ending the HIV epidemic: a plan for the United States. JAMA.

[40] Cabana MD, Rand CS, Powe NR, Wu AW, Wilson MH, Abboud PC, Rubin HR (1999). Why don't physicians follow clinical practice guidelines? A framework for improvement. JAMA.

[41] Hong B, O'Sullivan ED, Henein C, Jones CM (2019). Motivators and barriers to engagement with evidence-based practice among medical and dental trainees from the UK and Republic of Ireland: a national survey. BMJ Open.

[42] Michie S, van Stralen MM, West R (2011). The behaviour change wheel: a new method for characterising and designing behaviour change interventions. Implement Sci.

[43] Damschroder LJ, Aron DC, Keith RE, Kirsh SR, Alexander JA, Lowery JC (2009). Fostering implementation of health services research findings into practice: a consolidated framework for advancing implementation science. Implement Sci.

[44] Damschroder LJ, Reardon CM, Widerquist MAO, Lowery J (2022). The updated consolidated framework for implementation research based on user feedback. Implement Sci.

[45] Pratt Madeline C, Hill Samantha V, Elopre L, Simpson Tina, Lanzi Robin, Matthews Lynn T (2022). PrEP prescription for Black adolescent girls and young women in Alabama: findings from a survey of healthcare providers. J Int Assoc Provid AIDS Care.

[46] Fernandez ME, Ruiter RAC, Markham CM, Kok G (2019). Intervention mapping: theory- and evidence-based health promotion program planning: perspective and examples. Front Public Health.

[47] Garba RM, Gadanya MA (2017). The role of intervention mapping in designing disease prevention interventions: a systematic review of the literature. PLoS One.

[48] Scallan E, Davis S, Thomas F, Cook C, Thomas K, Valverde P, Kazanjian M, Byers T (2017). Supporting peer learning networks for case-based learning in public health: experience of the rocky mountain public health training center with the ECHO training model. Pedagogy Health Promot.

[49] Furlan AD, Zhao J, Voth J, Hassan S, Dubin R, Stinson JN, Jaglal S, Fabico R, Smith AJ, Taenzer P, Flannery JF (2019). Evaluation of an innovative tele-education intervention in chronic pain management for primary care clinicians practicing in underserved areas. J Telemed Telecare.

[50] Arora S, Kalishman S, Dion D, Som D, Thornton K, Bankhurst A, Boyle J, Harkins M, Moseley K, Murata G, Komaramy M, Katzman J, Colleran K, Deming P, Yutzy S (2011). Partnering urban academic medical centers and rural primary care clinicians to provide complex chronic disease care. Health Aff (Millwood).

[51] Essential sexual health questions to ask adolescents. National Coalition for Sexual Health.

[52] Sexual health and your patients: a provider's guide. National Coalition for Sexual Health.

[53] California Prevention Training Center Sexual history-taking toolkit: focus on chlamydia, gonorrhea and HIV in the family planning clinic. The National Network of STD Clinical Prevention Training Centers (NNPTC).

[54] (2022). Sexually transmitted diseases (STDs). A guide to taking a sexual history. Centers for Disease Control and Prevention (CDC).

[55] (2019). High school YRBS: Alabama 2019 and United States 2019 Results. Centre for Disease Control and Prevention.

[56] (2019). 2019 State of Alabama HIV surveillance annual report. Alabama Department of Public Health OoHPaC.

[57] (2019). Brief Facts on African-Americans and HIV in Alabama. Alabama Department of Public Health DoSHPaC.

[58] Cannon SM, Graber S, King HL, Hanashiro M, Averbach S, Moore DJ, Blumenthal J (2021). PrEP university: a multi-disciplinary university-based HIV prevention education program. J Community Health.

[59] Carnevale C, Zucker J, Womack JA, Dixon J, Cohall A, Sobieszczyk ME, Gordon P (2019). Adolescent preexposure prophylaxis administration: an education curriculum for health care providers. J Pediatr Health Care.

[60] Preexposure prophylaxis for the prevention of HIV infection in the United States - 2021 update: a clinical practice guideline. Centre for Disease Control and Prevention.

[61] (2018). Preexposure prophylaxis for the prevention of HIV infection in the United States 2017 update clinical practice guideline. Centers for Disease Control and Prevention: US Public Health Service.

[62] Wilson K, Beckett CG, Blaylock JM, Okulicz JF, Scott PT, Hakre S (2020). Provider knowledge gaps in HIV PrEP affect practice patterns in the US Navy. Mil Med.

[63] Seidman D, Carlson K, Weber S, Witt J, Kelly PJ (2016). United States family planning providers' knowledge of and attitudes towards preexposure prophylaxis for HIV prevention: a national survey. Contraception.

[64] Newman SD, Andrews JO, Magwood GS, Jenkins C, Cox MJ, Williamson DC (2011). Community advisory boards in community-based participatory research: a synthesis of best processes. Prev Chronic Dis.

[65] Pinto RM, Spector AY, Valera PA (2011). Exploring group dynamics for integrating scientific and experiential knowledge in community advisory boards for HIV research. AIDS Care.

[66] Powell BJ, Waltz TJ, Chinman MJ, Damschroder LJ, Smith JL, Matthieu MM, Proctor EK, Kirchner JE (2015). A refined compilation of implementation strategies: results from the expert recommendations for implementing change (ERIC) project. Implement Sci.

[67] Gui X, Chen Y, Zhou X, Reynolds TL, Zheng K, Hanauer DA (2020). Physician champions' perspectives and practices on electronic health records implementation: challenges and strategies. JAMIA Open.

[68] Kaasalainen S, Ploeg J, Donald F, Coker E, Brazil K, Martin-Misener R, Dicenso A, Hadjistavropoulos T (2015). Positioning clinical nurse specialists and nurse practitioners as change champions to implement a pain protocol in long-term care. Pain Manag Nurs.

[69] Rantz MJ, Zwygart-Stauffacher M, Flesner M, Hicks L, Mehr D, Russell T, Minner D (2012). Challenges of using quality improvement methods in nursing homes that "need improvement". J Am Med Dir Assoc.

[70] Ming K, Shrestha I, Vazquez A, Wendelborn J, Jimenez V, Lisha N, Neilands TB, Scott H, Liu A, Steward W, Johnson MO, Saberi P (2020). Improving the HIV PrEP continuum of care using an intervention for healthcare providers: a stepped-wedge study protocol. BMJ Open.

[71] NIMH Multisite HIV/STD Prevention Trial for African American Couples Group (2008). Eban HIV/STD risk reduction intervention: conceptual basis and procedures. J Acquir Immune Defic Syndr.

[72] Weiner BJ, Lewis CC, Stanick C, Powell BJ, Dorsey CN, Clary AS, Boynton MH, Halko H (2017). Psychometric assessment of three newly developed implementation outcome measures. Implement Sci.

[73] Aarons GA (2004). Mental health provider attitudes toward adoption of evidence-based practice: the evidence-based practice attitude scale (EBPAS). Ment Health Serv Res.

[74] Ariffin F, Chin KL, Ng C, Miskan M, Lee VK, Isa MR (2015). Are medical students confident in taking a sexual history? An assessment on attitude and skills from an upper middle income country. BMC Res Notes.

[75] Robins LS, Jackson JE, Green BB, Korngiebel D, Force RW, Baldwin L (2013). Barriers and facilitators to evidence-based blood pressure control in community practice. J Am Board Fam Med.

[76] Brown KM, Lindenberger JH, Bryant CA (2008). Using pretesting to ensure your messages and materials are on strategy. Health Promot Pract.

[77] Koenig CJ, Abraham T, Zamora KA, Hill C, Kelly PA, Uddo M, Hamilton M, Pyne JM, Seal KH (2016). Pre-implementation strategies to adapt and implement a veteran peer coaching intervention to improve mental health treatment engagement among rural veterans. J Rural Health.

[78] Miles MB, Huberman AM (1984). Qualitative data analysis: a sourcebook of new methods.

[79] Strauss A, Corbin J (1990). Basics of qualitative research.

[80] Patton M (2002). Qualitative Research & Evaluation Methods. 3rd ed.

[81] Owens C (2022). HIV pre-exposure prophylaxis awareness, practices, and comfort among urban and rural family medicine physicians. J Rural Health.

[82] Creger T, Burgan K, Turner WH, Tarrant A, Parmar J, Rana A, Mugavero M, Elopre L (2022). Using implementation mapping to ensure the success of prep optimization through enhanced continuum tracking (PrOTECT) AL-a structural intervention to track the statewide PrEP care continuum in Alabama. J Acquir Immune Defic Syndr.

[83] Elopre L, Williams A, Matthews L, Mugavero M, Kempf M (2021). PrEP 4 Her: Developing a Novel Strategy to Implement PrEP into Women's Health Care.

[84] Elopre L, Matthews LT, Rana A, Budhwani H (2022). CAMELLIA Cohort: A Longitudinal Study to Understand Sexual Health and Prevention Among Women in Alabama.

